# Using the behaviour change wheel to develop a tailored intervention to overcome general practitioners’ perceived barriers to referring insomnia patients to digital therapeutic sleepio

**DOI:** 10.1186/s12913-024-11384-3

**Published:** 2024-08-22

**Authors:** Ohoud Alkhaldi, Brian McMillan, John Ainsworth

**Affiliations:** 1 Division of Informatics, Imaging and Data Sciences, School of Health Sciences, Faculty of Biology, Medicine and Health, The University of Manchester, Oxford Road, Manchester, M13 9PM UK; 2 Health Information Management and Technology Department, College of Public Health, Imam Abdulrahman Bin Faisal University, Dammam, Saudi Arabia; 3 Centre for Primary Care and Health Services Research, Division of Population Health, Health Services Research and Primary Care, School of Health Sciences, Faculty of Biology, Medicine and Health, The University of Manchester, Manchester, M13 9PM UK; 4 NIHR Manchester Biomedical Research Centre, Manchester University Hospitals NHS Foundation Trust, Manchester Academic Health Science Centre, Manchester, UK

**Keywords:** COM-B, Behaviour change, Digital therapeutic, Insomnia

## Abstract

**Background:**

Digital therapeutic Sleepio has proven effective in improving sleep quality and decreasing symptoms of anxiety. The National Institute for Health and Care Excellence (NICE) guidance recommends Sleepio as an alternative treatment to usual sleep hygiene education and hypnotic medications. General practitioners (GPs) play a critical role in the adoption of digital therapeutics in patient care. Previous interventions did not adopt theoretical frameworks to systematically understand GPs behaviour toward referring patients to digital therapeutics.

**Objectives:**

This study aimed to report the systematic and comprehensive development of an intervention to encourage GPs to refer insomnia patients to Sleepio, using the Behaviour Change Wheel (BCW).

**Methods:**

The eight steps outlined in the BCW were followed to develop an intervention. The Capability Opportunity Motivation-Behaviour Self-Evaluation Questionnaire (COM-B-Qv1) was adopted to understand GPs perceived facilitators and barriers to refer insomnia patients to Sleepio. The Behaviour Change Technique Taxonomy Version 1 (BCTv1) was thereafter used to identify possible strategies that could be used to facilitate changes in GPs’ behaviour in relation to Sleepio.

**Results:**

The BCW design process resulted in the identification of five intervention functions, three policy categories and five behaviour change techniques (BCTs) as potential active components for an intervention. The intervention includes providing GPs with an orientation about using Sleepio to improve their knowledge and confidence, sending visual reminders to GPs to recommend Sleepio to their patients, providing ongoing technical support.

**Conclusion:**

The BCW can be successfully applied through a systematic process to understand the drivers of GPs’ behaviour and to develop an intervention that can encourage them to refer insomnia patients to Sleepio.

**Supplementary Information:**

The online version contains supplementary material available at 10.1186/s12913-024-11384-3.

## Introduction

Insomnia is a health condition that causes difficulty to sleep or a total lack of sleep [1]. It is estimated to affect one in three adults in the UK, and higher rates are associated with being female, being older and the presence of comorbidities [2, 3]. Insomnia is associated with a significant economic burden due to absenteeism, reduced productivity and impaired cognition and mood [4].

Guidelines recommend cognitive behavioural therapy for insomnia (CBT-I) for the treatment of insomnia rather than pharmacologic and drug therapy due to reported side effects, such as the possibility of developing long-term tolerance and addiction [5]. Studies have shown that benzodiazepine use is a significant risk factor for fall-related accidents among older adults [6, 7].

CBT-I is a psychological treatment that guides patients to change their sleep-related behaviour through a series of techniques in weekly courses that last for five weeks [8]. However, there are difficulties surrounding CBT-I that limit its prescription to patients, such as issues with accessibility and costs, and poor response [8].

Sleepio is a digital therapeutic that uses CBT-I and can be accessed through self-referral or general practitioner (GP) referrals. The programme has proven effective in improving sleep quality and decreasing symptoms of anxiety and depression [9]. In an ambitious attempt to integrate digital therapeutics into patient care, NHS Scotland made Sleepio free of charge to all residents of Scotland in October 2020 [10].

For GPs, recommending digital therapeutics differs from the standard practice of prescribing medications. Evidence of interventions to improve GPs’ adoption of mobile health (mHealth) apps and/ or digital therapeutics is limited. One interrupted time series study aimed to evaluate the cost-effectiveness of Sleepio in primary care settings in England. As part of the study, attention was given to selected GP practices that involved implementing training and digital prompts for GPs and distributing patient-centred resources and awareness material to practices, along with tailored support [11]. Some of these implementation strategies targeted patients, while others were tailored to healthcare professionals (HCPs). The study found that a lower level of uptake was observed in areas that lacked involvement in implementation strategies, which suggests that for future work, the impact of resources on Sleepio referrals should be assessed. In another study, the intervention included 40 min of Sleepio training for clinical staff, a protocol for all clinical assessments and a website with logistical tools to assist staff [12]. There were no measures of the intervention’s impact on GPs’ referral behaviour. Therefore, this paper describes the development of an intervention to encourage GPs to refer insomnia patients to Sleepio.

### The theory

To understand the current behaviour and select the best interventions that will most likely be beneficial, the behaviour change wheel (BCW) will be used [13]. This framework provides a comprehensive approach to identifying sources of behaviour and classifying them into the capability, opportunity, motivation, and behaviour (COM-B) model. This model helps to understand the behaviour of interest and select the behaviour change techniques (BCTs) most likely to be effective. The COM-B model is designed to capture factors that affect the target behaviour (physical capability, psychological capability, physical opportunity, social opportunity, reflective motivation and automatic motivation).

The theoretical domain framework (TDF) is a comprehensive framework used in behaviour science and implementation research to determine the key areas that affect behaviour change [14]. The TDF is used in conjunction with the COM-B model if a more detailed understanding of the behaviour is required.

Several studies in the literature have used the BCW framework to design interventions targeting GPs. One study aimed to assess the effectiveness of a COM-B-based intervention in promoting physical activity among GPs and their patients [15]. Another study explored the role of GPs in facilitating behaviour change using the TDF and BCW [16]. While no studies in the literature focused on the use of BCW with GPs in the context of mHealth, the findings of other studies provided insights that BCW could be applied to other disciplines. Overall, the use of the BCW in conjunction with GPs has proven to be valuable in understanding and addressing barriers to behaviour change.

## Methods

### Aim

This study aimed to report the systematic and comprehensive development of an intervention to encourage GPs to refer insomnia patients to Sleepio, using the Behaviour Change Wheel (BCW).

### Design

The intervention development followed the BCW developed by Michie et al. [13]. The process consists of eight steps, as follows:

#### Step 1: Define the problem

We reviewed the literature regarding barriers to and facilitators of prescribing mHealth apps in general from the point of view of HCPs. By doing this, we aimed to cover all factors that affect behaviour, including environmental, physical and social contexts.

#### Step 2: Select the target behaviour

In this step, all possible factors that affect behaviour and could be targeted in the intervention were investigated. To determine the target behaviour, the literature on GPs’ use of evidence-based digital therapeutics in the UK was reviewed.

#### Step 3: Specify the target behaviour

After selecting the target behaviour, specific details were identified, such as who would perform it, what must be done to achieve the desired change and when, where and how often it needed to be done.

#### Step 4: Identify what needs to change

This step involved behavioural analysis using the COM-B model (capability, opportunity and motivation to recommend a digital therapeutic) to identify which component of the model needed to change to achieve the desired results. The COM-B self-evaluation questionnaire (COM-B-Qv1) was used to understand what it would take for participants to change their behaviour.

##### Survey development and analysis

GPs in Scotland were invited to take part in the survey because Sleepio is only available for GP referrals in Scotland. The survey was prepared using Qualtrics (www.qualtrics.com) and distributed online, mainly through the primary care research network in Scotland. The NHS Research Scoltand (NRS) Primary Care Network is a unit that supports researchers in recruiting participants using electronic databases [17].

The survey (Supplementary file 1) consisted of 27 items, including questions relating to demographics, GPs’ behaviour in terms of recommending Sleepio, and questions based on the COM-B self-evaluation questionnaire (COM-B-Qv1), which is recommended for collecting data during the BCW intervention development process [13].

The (COM-B-Qv1)18-item scale had a Cronbach’s alpha of 0.910, with 6-item subscale alphas of 0.793 for capabilities, 0.853 for opportunities and 0.812 for motivations. The full scale is available in the supplementary material (Supplementary File 1 includes the survey questions).

The survey was pilot-tested among 12 GPs and 5 health informatics PhD students at the University of Manchester, and feedback was sought about survey items, the format and the time taken for completion before finalising the survey.

GPs were provided with information about the survey (e.g. background and importance of the study, purpose of the study, potential benefits of taking part, and how their personal information would be stored and processed) via an information sheet on the online survey platform. GPs provided consent by selecting the checkbox to confirm that they agreed with the information provided and were happy to participate in the study. Participants received no compensation for completing the questionnaire. The estimated time to complete the survey 4–7 min and was available between available between February and April 2023 (75 days).

Questionnaire data were analysed using IBM SPSS Statistics V.25, including the descriptive data analysis of participant characteristics. The responses from the 5-point Likert scales were combined to create a 3-point scale by combining ‘agree’ and ‘strongly agree’ and ‘disagree’ and ‘strongly disagree’. The frequency and percentage of each COM-B statement response were calculated.

#### Step 5 & 6: Identify intervention functions and select policy categories

Based on the findings of the questionnaire, we used the BCW to select the most appropriate intervention functions to design the intervention.

#### Step 7: Select behaviour change techniques

Michie et al. identified 93 possible BCTs, each linked to intervention functions [18]. In this step, we selected the most effective techniques to produce a successful change in GPs’ prescribing behaviour.

#### Step 8: Determine the mode of delivery

After selecting BCTs, it is important to consider the mode or modes of delivery most appropriate for the target behaviour. In this step, we considered the difficulty of engaging GPs in research for reasons such as workload and lack of time.

### Results

#### Step 1: Define the problem

Several studies have revealed that HCPs’ lack of knowledge and awareness of available apps are major barriers to incorporating them into patient care [19–21]. To overcome these barriers, several studies have emphasised the need to design training for GPs and other allied health professionals to improve their knowledge of the importance of prescribing mHealth apps to patients with long-term conditions [20–23]. The cost of using mobile health apps is also a concern for some GPs [24].

#### Step 2: Select the target behaviour

By reviewing the literature in the UK, we found that Sleepio is the first digital therapeutics to receive NICE guidance as an effective digital treatment for insomnia before prescribing sleeping drugs or sleep hygiene. Many studies concluded Sleepio is more effective than usual treatment in reducing symptoms of insomnia in adults [25, 26]. However, GPs behaviour toward Sleepio remains unknown. We decided that the intervention should target GPs [27]. The study is expected to help GPs incorporate digital therapeutics into patient care and improve their confidence in recommending evidence-based apps.

#### Step 3: Specify the target behaviour

After selecting the target behaviour, further details were determined by answering the questions in Table 1. GPs can refer insomnia patients to Sleepio if Sleepio is deemed the right treatment option for them.

**Table 1:** Specifying Target Behaviour – B.3

Target behaviour	GPs incorporating digital therapeutics in primary care to treat insomnia
Who needs to perform the behaviour?	GPs in Scotland
What does the person need to do differently?	Refer insomnia patients to Sleepio
When?	If Sleepio is the right treatment for their patients
Where?	Primary care
How often?	Always
With whom?	Insomnia patients

#### Step 4: Identify what needs to change

To identify what needed to change, we surveyed GPs in Scotland about their attitudes towards referring insomnia patients to Sleepio. Seventy participants responded to the questionnaire. Five questionnaires were incomplete, leaving sixty-five participants with a full set of data. Table 2 presents the participants’ demographic data.

**Table 2:** Baseline characteristics of participants

Characteristics	*N* (%)
Age (years)	
26–35 36–45 46–55 56–65 > 65	6 (9.2) 24 (36.9) 24 (36.9) 9 (13.8) 2 (3.1)
Gender	
Male Female Prefer not to say	16 (24.6) 48 (73.8) 1 (1.5)
GP Practice location	
Ayrshire and Arran Borders Dumfries and Galloway Fife Forth Valley Grampian Greater Glasgow and Clyde Lanarkshire Lothian Orkney Oxfordshire Berkshire Other	3 (4.6) 1 (1.5) 4 (6.2) 1 (1.5) 15 (23.1) 4 (6.2) 15 (23.1) 8 (12.3) 8 (12.3) 1 (1.5) 1 (1.5) 2 (3.1) 2 (3.1)
Years of work experience	
< 5 years 6–10 years 11–15 years 16–20 years > 20 years	14 (21.5) 8 (12.3) 7 (10.8) 12 (18.5) 24 (36.9)
Received training about digital therapeutics	
No Yes	55 (84.6) 10 (15.4)
Aware of Sleepio	
No Yes	16 (24.6) 49 (75.4)
How often do you prescribe Sleepio?	
Always Most of the time About half of the time Sometimes Never	4 (6.2) 27 (41.5) 8 (12.3) 14 (21.5) 12 (18.5)

**Fig. 1:**
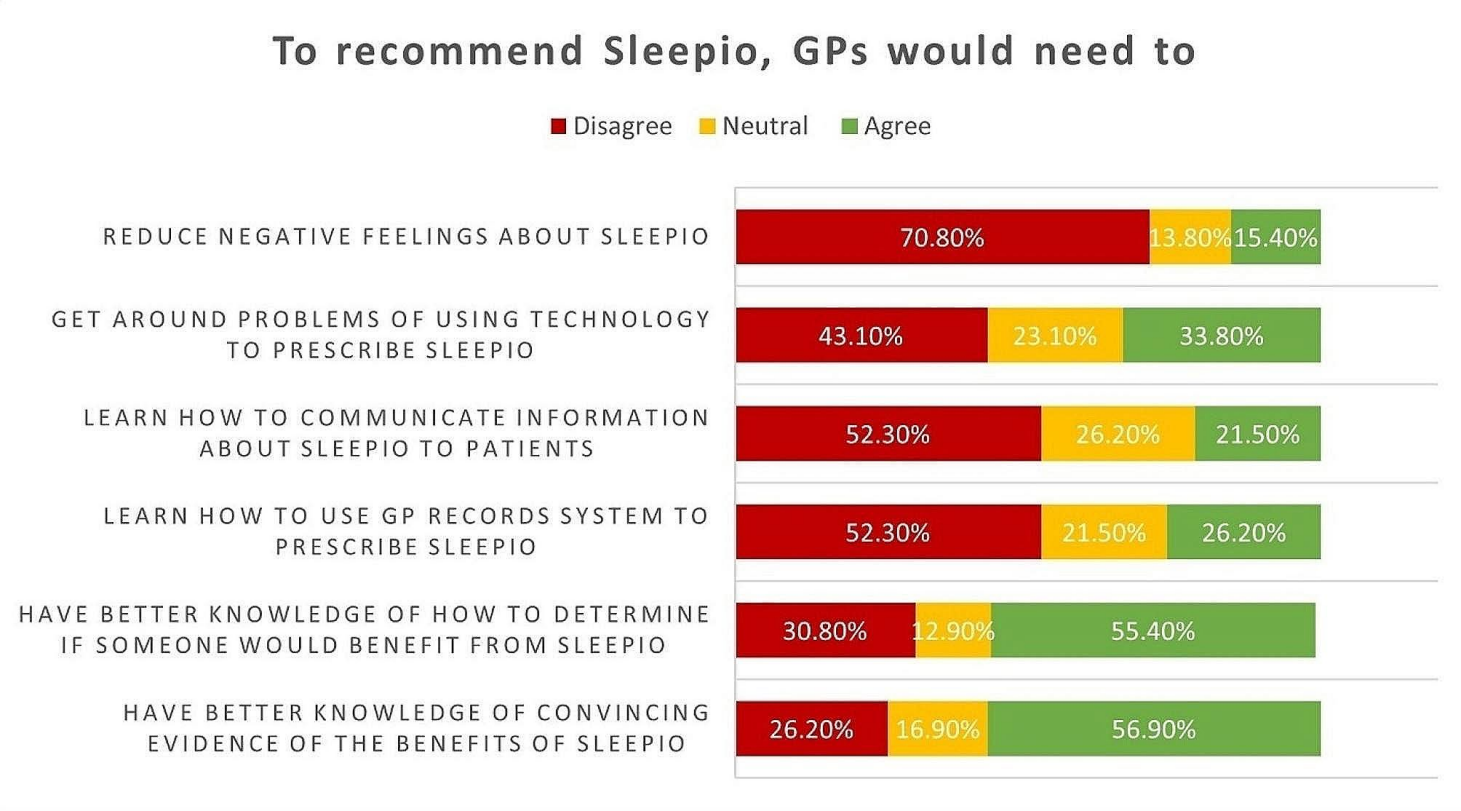
GPs’ Responses to Capability Statements

**Fig. 2:**
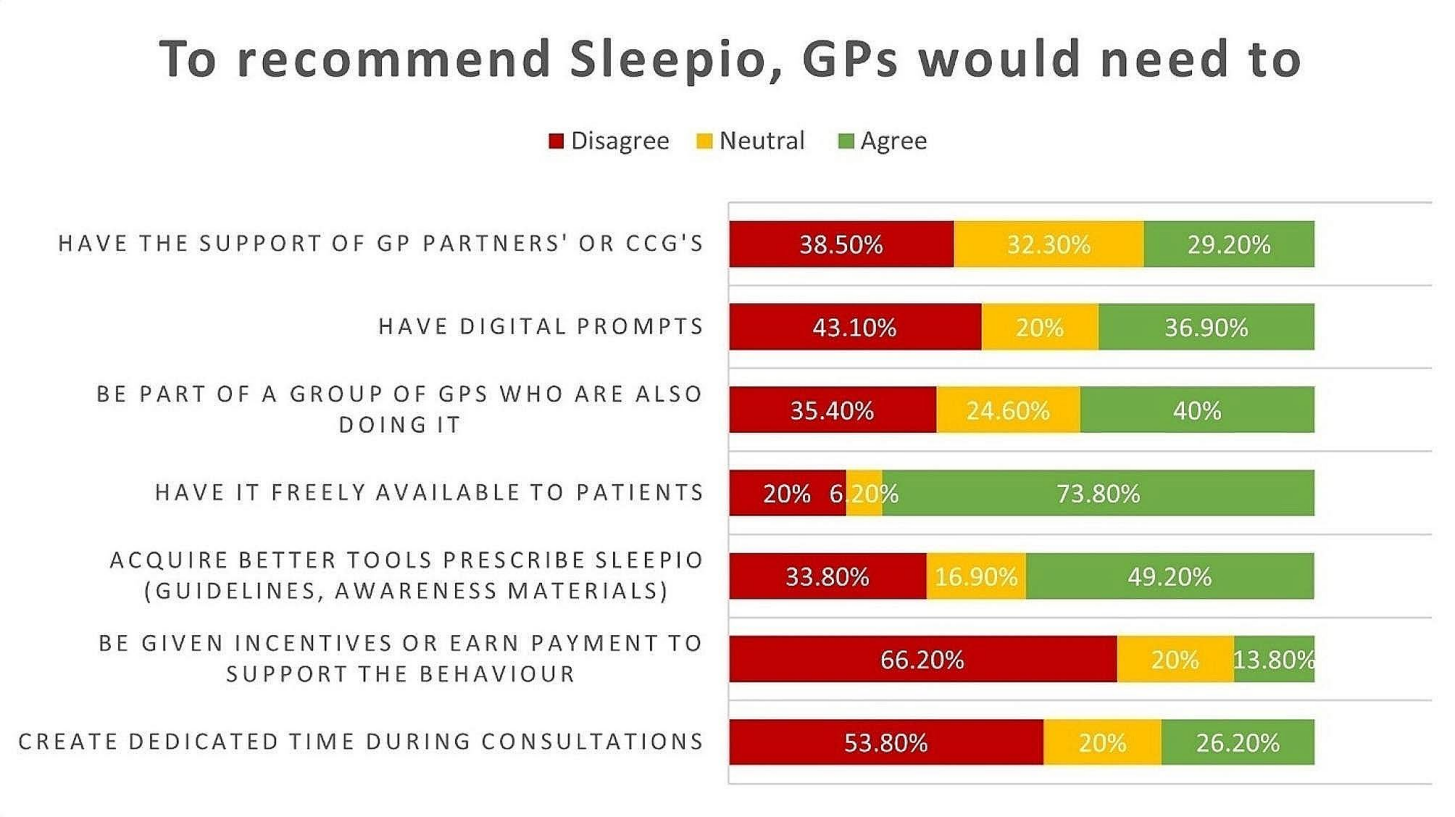
GPs’ Responses to Opportunity Statements

**Fig. 3:**
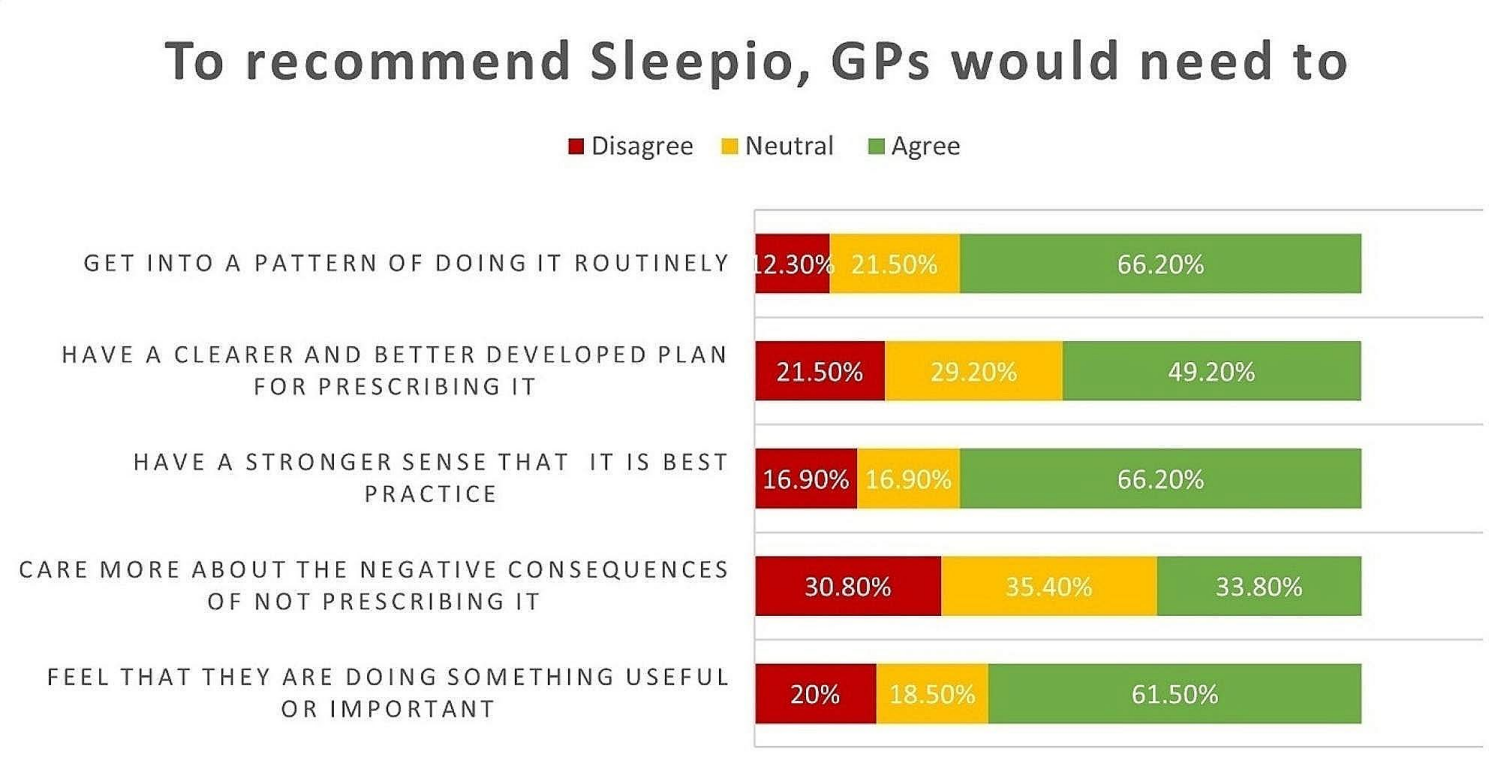
GPs’ Responses to Motivation Statements

Participants were asked to rate the extent to which they agreed with each statement. GPs’ ratings on questionnaire statements in each COM-B domain about what would make them recommend Sleepio to their patients are illustrated in Figs. 1, 2 and 3.

From the survey, it was determined that the intervention needed to target most components of the COM-B model, with a strong focus on psychological capability, physical opportunity and automatic and reflective motivation.

##### Psychological capability

About 57% (37/65) of participants reported that knowing the clinical evidence behind the digital therapeutic Sleepio would encourage them to offer it to their patients. Around 56% (36/65) of respondents reported knowing how to determine whether patients would benefit from Sleepio by assessing its clinical suitability and patients’ ability to engage with it. This highlights knowledge, memory, attention and decision processes as important in the TDF to be addressed in the intervention.

##### Physical opportunity

The cost of digital therapeutics was significantly associated with a reduced likelihood of referring insomnia patients to Sleepio. Participants reported that they would refer patients to Sleepio if it was made freely available to them. Linking that with TDF domains, it was found that environmental context and resources were important in encouraging GPs to recommend Sleepio.

##### Automatic motivation

GPs reported that making changes to their prescribing habits would facilitate more frequent referrals to Sleepio. GPs need to discuss and recommend Sleepio for any patients who complain about their sleep patterns before prescribing medications. Reinforcement was found to be a crucial TDF domain for inclusion in the intervention.

##### Reflective motivation

GPs who believe that Sleepio can assist insomnia patients in regaining normal sleeping patterns are more likely to refer them to Sleepio. They need to believe that recommending Sleepio is the best practice. Therefore, targeting beliefs about the outcomes of the intervention would work as a facilitator for changing the target behaviour.

#### Step 5 & 6: Identify intervention functions and select policy categories

The COM-B behavioural analysis identified five intervention functions: education, training, environmental restructuring, enablement and persuasion. Policy categories that matched our intervention functions included communication/marketing (for instance, using verbal, electronic communication or flyers to improve knowledge of referring patients to Sleepio and health consequences of using Sleepio), guidelines (examples of which include informing GPs of steps for offering Sleepio) and environmental/social planning (e.g., sending a visual reminder to GPs to recommend Sleepio through emails) (Table 3).

**Table 3:** Linking between COM-B model, TDF domains, intervention functions, policy categories and BCTs

Barriers to referring insomnia patients to Sleepio	COM-B components	Theoretical domains framework	Intervention functions	Policy categories	Behaviour change techniques	Description of behaviour change techniques within the intervention
GPs need to know more about why it is important, e.g. have a better knowledge of convincing evidence of the benefits of Sleepio.	Psychological capability	Knowledge	Education	Communication/ Marketing	Information about health consequences	Introduce Sleepio and the evidence base for using digital therapeutics (Sleepio) to treat insomnia and inform GPs how to determine if someone would benefit from Sleepio using
GPs need to know more about how to do it, e.g. have a better knowledge of how to determine if someone would benefit from Sleepio.	Psychological capability	Memory, attention and decision processes	Training, Environmental restructuring Enablement	Guidelines Environmental/Social planning	• Instruction on how to perform a behaviour • Prompts/ Cues	• Provide GPs steps for offering Sleepio using training materials • Introduce or define environmental or social stimulus to promote the behaviour by sending a visual reminder to GPs to recommend Sleepio through emails
GPs want it to be more easily accessible, e.g. have it freely available to patients.	Physical opportunity	Environmental context and resources	Environmental restructuring	Environmental/Social Planning	Adding objects to the environment	Sleepio is free for prescription in Scotland but GPs are unaware of this. Therefore, by implementing this intervention, GPs will be aware of Sleepio and how to refer insomnia patients to Sleepio
GPs need to develop a habit of doing it, e.g. get into a pattern of doing it routinely.	Automatic motivation	Reinforcement	Training	Guidelines Environmental/Social Planning	Self-monitoring of behaviour	Report the number of referrals to Sleepio through short surveys every two weeks of the intervention
GPs must believe that it is a good thing to prescribe, e.g. have a stronger sense that it is best practice.	Reflective motivation	Beliefs about consequences	Persuasion	Communication/ Marketing	Credible source	Ask GPs to review orientation presentation that highlights cognitive behavioural therapy (CBT) as the recommended first-line treatment for insomnia

#### Step 7: Select behaviour change techniques

In total, six behaviour change techniques were selected. The main BCTs selected for encouraging GPs to refer insomnia patients to Sleepio were information about health consequences, instruction on how to perform behaviour, prompts/cues, adding objects to the environment, self-monitoring of behaviour and credible sources.

#### Step 8: Determine the mode of delivery

GPs reported concerns that changing their work habits might cause an extra burden on their daily work schedules. Therefore, to ensure GPs’ engagement, the intervention is designed to be delivered online on an individual level and can be accessed via computers at a convenient time.

## Discussion

This study provides a structured and detailed example of how to design an intervention to target GPs in primary care using the BCW. The BCW framework was used to systematically understand the target behaviour before the intervention was designed in terms of changes to capability, opportunity and motivation (the COM-B system).

GPs reported that they would recommend Sleepio if they had greater capabilities especially in psychological capability domain. This included having better knowledge of convincing evidence of the benefits of Sleepio and knowing how to determine if someone would benefit from Sleepio. This is in line with previous recommendations regarding the need to design training for GPs and other allied health professionals for prescribing mHealth apps to patients with long-term conditions [19–21].

However, the findings of this study showed that providing GPs with information about Sleepio may not be enough to produce a change in the target behaviour. The survey results indicated that in relation to opportunity, GPs would recommend Sleepio more often if it was freely available to patients. This indicates a potential lack of awareness among GPs regarding Sleepio’s existing availability for GP referral to all adults in Scotland. It was found that this factor was the overarching barrier to referring patients to digital therapeutic Sleepio. In line with the findings of the current study, a previous study reported that the cost of apps was significantly associated with the likelihood of prescribing digital health technologies, suggesting that as cost increases, the rate of digital health technology prescriptions falls [24]. Moreover, a review of an intervention study found that while providing education and skills training is likely to improve nutritionists’ self-efficacy, having the app easily and freely integrated into dietetic care is essential to influence the prescribing of apps [28].

In relation to motivation to recommend Sleepio, GPs reported needing to develop a pattern of doing it routinely and have a stronger sense that it is best practice. GPs’ responses concerning motivation reflected that while they had a strong motivation to incorporate digital therapeutics into patient care or develop habits of recommending Sleepio to their patients, they may not have done so primarily due to the perceived difficulty of accessing Sleepio (opportunity) or a lack of knowledge (capability). Therefore, addressing barriers related to opportunity and capability is likely to produce changes in motivation [29].

In a future intervention, a number of BCTs will be included to maximise successful changes in the target behaviour, such as the inclusion of evidence and scientific rationale for using a digital therapeutic (Sleepio) to treat insomnia, providing GPs with clear steps for offering Sleepio and making sure that GPs are aware that Sleepio is made free to all adults in Scotland. Additionally, to address GPs concerns about the increased workload and time demands when apps are integrated into daily work activities [30–32], interventions should be delivered online. This will allow them to access the training materials at their convenience. We are in the process of designing an intervention and piloting it to improve GP referrals to Sleepio to treat insomnia patients as an alternative to usual treatments.

### Strengths and limitations

This study has a number of strengths. To the authors’ knowledge, this is the first study to investigate the influence of both behavioural and environmental determinants on GPs’ referral attitudes towards the digital therapeutic Sleepio.

The BCW provided a systematic approach to achieving a better understanding of GPs’ perceived barriers to incorporating this digital therapeutic in routine care, and it was found effective in designing an intervention to target GPs’ needs. Furthermore, the COM-B self-evaluation questionnaire (COM-B-Qv1) provided information on self-reported behavioural determinants of Sleepio referrals, which enabled participants to consider a wide spectrum of factors relating to these (i.e. the capability, opportunity and motivation subscales).

With regard to study limitations, we recruited GPs in Scotland only, which reduces the study’s generalisability. Generalisability was also affected by the limited sample size, although the study was advertised using a research network in Scotland. To enhance generalisability, future studies should combine multiple approaches to increase GP participation, such as using social media and targeting GPs in conferences. A possible source of bias is that GPs who were interested in mobile health and cognitive behaviour therapy were more likely to be motivated to complete the survey, leading to self-selection bias.

## Conclusions

This study identified a number of intervention components that can be applied to encourage GPs to recommend Sleepio for CBT-I treatment as an alternative to medications for insomnia. This study highlighted the importance of interventions targeting multiple levels of behaviour to produce change. Six BCTs were identified as core methods that affect psychological capability, physical opportunity, automatic motivation and reflective motivation of GPs’ behaviour with regard to referring patients to Sleepio. Future studies should evaluate the feasibility of an intervention based on the findings reported here.

### Electronic supplementary material

Below is the link to the electronic supplementary material.

Supplementary Material 1

## Data Availability

The authors confirm that the data supporting the findings of this study are available within the article and its supplementary materials.

## Abbreviations

BCTs: Behaviour Change Techniques
BCTv1: Behaviour Change Technique Taxonomy Version 1
BCW: Behaviour Change Wheel
CBT-I: Cognitive Behavioural Therapy for Insomnia
COM-B: Capability, Opportunity, Motivation, and Behaviour model
COM-B-Qv1: Capability Opportunity Motivation-Behaviour Self-Evaluation Questionnaire
GPs: General practitioners
HCPs: Healthcare Professionals
NICE: National Institute for Health and Care Excellence
NRS: The NHS Research Scotland
TDF: The theoretical domain framework
