# Supplementary material for: Using the behaviour change wheel to develop a tailored intervention to overcome general practitioners’ perceived barriers to referring insomnia patients to digital therapeutic sleepio
Source: BMC Health Serv Res. 2024 Aug 22;24:967. doi: 10.1186/s12913-024-11384-3 (PMC11340175; doi:10.1186/s12913-024-11384-3)
Supplement: Supplementary file 1 — Supplementary Material 1 [file 12913_2024_11384_MOESM1_ESM.docx]

Appendix A. Supplementary file

**Survey questions:**

**Part A:**

1. Age in years:

- <26
- 26 – 35
- 36– 45
- 46 – 55
- 56 – 65
- > 65

1. Gender:

- Male
- Female
- Prefer not to say

1. GP Practice Location:

- Ayrshire and Arran
- Borders
- Dumfries and Galloway
- Fife
- Forth Valley
- Grampian
- Greater Glasgow and Clyde
- Highland
- Lanarkshire
- Lothian
- Orkney
- Shetland
- Tayside
- Western Isles
- Buckinghamshire
- Oxfordshire
- Berkshire
- Other: ___________________

1. Years of work experience as a GP in general practice:

- Five years or less
- 6 – 10 years
- 11-15 years
- 16- 20 years
- More than 20 years

1. Have you completed any training about using digital therapeutics in patient care?

- Yes
- No

1. Were you aware before starting this questionnaire that the digital therapeutic (Sleepio) has been recommended by NICE as an option for treating insomnia?

- Yes
- No

1. How often do you prescribe Sleepio for patients with symptoms of Insomnia?

- Always
- Most of the time
- About half of the time
- Sometimes
- Never

**Part B: COM-B items (5-point Likert scale)**

The following section asks questions about factors that facilitate GPs’ readiness to recommend Sleepio as an alternative to standard care.

Please rate on a scale of 1 to 5 how strongly you agree or disagree with the following statements.

A. Capability

**"For me to prescribe Sleepio to patients with insomnia, I would have to... "**

|  | Strongly disagree  (1) | Disagree  (2) | Neutral  (3) | Agree  (4) | Strongly Agree  (5) |
| --- | --- | --- | --- | --- | --- |
| Know more about why it is important, e.g. have a better knowledge of convincing evidence of the benefits of Sleepio | **⃝** | **⃝** | **⃝** | **⃝** | **⃝** |
| Know more about how to do it, e.g. have a better knowledge of how to determine if someone would benefit from Sleepio | **⃝** | **⃝** | **⃝** | **⃝** | **⃝** |
| Have better technological skills, e.g. learn how to use GP records system to prescribe Sleepio | **⃝** | **⃝** | **⃝** | **⃝** | **⃝** |
| Have better communication skills, e.g. learn how to communicate information about Sleepio to patients | **⃝** | **⃝** | **⃝** | **⃝** | **⃝** |
| Overcome technological limitations, e.g. to get around problems of using technology to prescribe Sleepio | **⃝** | **⃝** | **⃝** | **⃝** | **⃝** |
| Overcome my preconceptions about Sleepio, e.g. reduce negative feelings about Sleepio | **⃝** | **⃝** | **⃝** | **⃝** | **⃝** |

B. Opportunity

**"For me to prescribe Sleepio to patients with insomnia, I would have to... "**

|  | Strongly disagree  (1) | Disagree  (2) | Neutral  (3) | Agree  (4) | Strongly Agree  (5) |
| --- | --- | --- | --- | --- | --- |
| Have more time to do it, e.g. create dedicated time during consultations | **⃝** | **⃝** | **⃝** | **⃝** | **⃝** |
| Have more funding, e.g. be given incentives or earn payment to support the behaviour | **⃝** | **⃝** | **⃝** | **⃝** | **⃝** |
| Have the necessary materials, e.g. acquire better tools to prescribe Sleepio (guidelines, awareness materials) | **⃝** | **⃝** | **⃝** | **⃝** | **⃝** |
| Have it more easily accessible, e.g. have it freely available to patients | **⃝** | **⃝** | **⃝** | **⃝** | **⃝** |
| Have more people around me doing it, e.g. be part of a group of GPs who are doing it | **⃝** | **⃝** | **⃝** | **⃝** | **⃝** |
| Have more triggers to prompt me, e.g. have digital prompts for GPs | **⃝** | **⃝** | **⃝** | **⃝** | **⃝** |
| Have more support from others, e.g. have my GP partners’ or CCG’s support | **⃝** | **⃝** | **⃝** | **⃝** | **⃝** |

C. Motivation

**"For me to prescribe Sleepio to patients with insomnia, I would have to... "**

|  | Strongly disagree | Disagree | Neutral | Agree | Strongly Agree |
| --- | --- | --- | --- | --- | --- |
| Feel that I want to do it enough, e.g. feel that I am doing something useful or important | **⃝** | **⃝** | **⃝** | **⃝** | **⃝** |
| Feel that I need to do it enough, e.g. care more about the negative consequences of not prescribing it | **⃝** | **⃝** | **⃝** | **⃝** | **⃝** |
| Believe that it would be a good thing to do, e.g. have a stronger sense that it is best practice | **⃝** | **⃝** | **⃝** | **⃝** | **⃝** |
| Develop better plans for doing it, e.g. have a clearer and better developed plan for prescribing it | **⃝** | **⃝** | **⃝** | **⃝** | **⃝** |
| Develop a habit of doing it, e.g. get into a pattern of doing it routinely | **⃝** | **⃝** | **⃝** | **⃝** | **⃝** |

- Please select the appropriate box below if you want your contact information saved so that study researchers can contact you in the future to invite you to participate in follow-up research on this project.

Yes, I would be happy to be contacted in this way (please ENTER a contact email address)

ꓫ_________________________________________________

No, I would not like to be contacted

- Do you have any other comments?

_____________________________________________________________________________

_____________________________________________________________________________

Thank you for completing the questionnaire!
